# Special Issue on Metabolism and Metabolite Markers in Type 2 Diabetes

**DOI:** 10.3390/metabo12070642

**Published:** 2022-07-13

**Authors:** Jan W. Eriksson

**Affiliations:** Department of Medical Sciences, Clinical Diabetology and Metabolism, Uppsala University, 75185 Uppsala, Sweden; jan.eriksson@medsci.uu.se

It is well recognized that type 2 diabetes poses a major and increasing public health challenge, and presently, almost 500 million individuals around the globe are diagnosed with this condition. It has a complex pathobiology, and typically evolves in the setting of combined defects in insulin secretion and insulin action. Many underlying mechanisms contribute to these perturbations, and they interact in a complex interplay involving genetic, epigenetic, neurohormonal, dietary, behavioral, and environmental factors. Fortunately, treatment tools are now available that can help patients to achieve appropriate glucose control and to lower their risk for comorbidities and premature death [1,2]. For example, in patients with type 2 diabetes and high cardiovascular risk, SGLT2 inhibitors have delivered strong benefits to prevent events of heart and renal failure as well as cardiovascular death. In such patients, several GLP-1 receptor agonists were shown to reduce the risk of atherosclerotic cardiovascular events and cardiovascular death. Moreover, emerging dual receptor agonists, in particular targeting GIP together with GLP-1 receptors, appear very promising. Besides glucose control, obviously other cardiometabolic risk factors need to be addressed, including hypertension, dyslipidemia, central obesity, and smoking. When all known risk factors are appropriately managed, the risk for both cardiovascular and microvascular complications is markedly reduced [3,4].

Despite the expanding treatment toolbox, there is, in reality, still a high risk which remains for comorbidities and premature death among people with type 2 diabetes [4,5]. To effectively mitigate such risks, we need a better basic disease understanding, improved tools for risk assessment and, most likely, novel pharmacological and nonpharmacological interventions. This will require further characterization of important metabolic pathways and biomolecules involved in type 2 diabetes and its comorbidities. Eventually, such efforts may support the advancement of precision medicine in type 2 diabetes, which may provide innovative and individualized tools for patient stratification, prevention, and therapy. This Special Issue of *Metabolites* focuses on advances in the field of metabolic regulation and dysregulation relevant to the development, progression, and prevention or reversal of type 2 diabetes and its comorbidities. The collection of review and original articles from seven different countries covers several aspects of metabolic pathways and markers in type 2 diabetes.

With respect to disease mechanisms, one of the papers discusses the impact of bacterial and fungal gut microbiota in the metabolic syndrome, and another one reports alterations in levels of essential trace elements such as copper, iron, and zinc in the development of type 2 diabetes. Pharmacological mechanisms involved in type 2 diabetes development are also discussed. Thus, there is a study on HMG-CoA reductase activity, cholesterol levels, and the diabetogenic effect of statin treatment. Additionally, adverse metabolic effects of antipsychotic drugs are presented, and endoplasmic reticulum stress in pancreatic beta cells is found after olanzapine exposure but could be prevented by a bile acid. When it comes to interventions, a meta-analysis of trials addressing supplementation with omega-3 polyunsaturated fatty acids proposes improvements in glucose and lipid control in T2D. There is also a review on plant-derived phenolic compounds for the prevention of T2D. Finally, another review paper elucidates the mechanisms of physical activity to reverse insulin resistance.

I hope you will enjoy this Special Issue on metabolic dysregulation and markers in type 2 diabetes.

## Data Availability

Not applicable.

## References

[1] Lin D.S., Lee J.K., Hung C.S., Chen W.J. (2021). The efficacy and safety of novel classes of glucose-lowering drugs for cardiovascular outcomes: A network meta-analysis of randomised clinical trials. Diabetologia.

[2] North E.J., Newman J.D. (2019). Review of cardiovascular outcomes trials of sodium-glucose cotransporter-2 inhibitors and glucagon-like peptide-1 receptor agonists. Curr. Opin. Cardiol..

[3] Gaede P., Vedel P., Larsen N., Jensen G.V., Parving H.H., Pedersen O. (2003). Multifactorial intervention and cardiovascular disease in patients with type 2 diabetes. N. Engl. J. Med..

[4] Rawshani A., Rawshani A., Franzén S., Sattar N., Eliasson B., Svensson A.M., Zethelius B., Miftaraj M., McGuire D.K., Rosengren A. (2018). Risk Factors, Mortality, and Cardiovascular Outcomes in Patients with Type 2 Diabetes. N. Engl. J. Med..

[5] Norhammar A., Bodegard J., Nystrom T., Thuresson M., Nathanson D., Eriksson J.W. (2019). Dapagliflozin and cardiovascular mortality and disease outcomes in a population with type 2 diabetes similar to that of the DECLARE-TIMI 58 trial: A nationwide observational study. Diabetes Obes. Metab..

